# Status asthmaticus with pseudo-dextrocardia, complicated by iatrogenic tension pneumothorax

**DOI:** 10.11604/pamj.2016.24.31.9552

**Published:** 2016-05-09

**Authors:** Poobalan Naidoo

**Affiliations:** 1 Khan Regional Hospital, University of Kwazulu-natal, Nelson Mandela College of Medicine, Department of Internal Medicine, Durban, South Africa

**Keywords:** Pneumothorax, pseudo-dextrocardia, status asthmaticus

## Image in medicine

A 25-year old female, with a background of asthma, presented with acute shortness of breath. On examination the patient was in severe respiratory distress with expiratory wheezes. She was nebulized with ipratropium and fenoterol. A chest x-ray revealed pseudo-dextrocardia and air trapping (A). She did not respond to inhaled bronchodilator therapy. Intravenous hydrocortisone and magnesium sulphate were administered. The patient deteriorated and was subsequently ventilated and intravenous aminophylline initiated. After intubation and ventilation, a central venous line was inserted. The patient deteriorated immediately after central line placement and an arterial blood gas showed type 2 respiratory failure with respiratory acidosis. The repeat x-ray revealed a right sided tension pneumothorax with displacement of the mediastinum to the left, incorrect placement of the central line, endotracheal tube and nasogastric tube (B). The central line and nasogastric tube were removed and an intercostal drain was inserted. The endotracheal tube was adjusted. The pneumothorax resolved and the patient made an uneventful recovery. The patient was mechanically ventilated, had severe airway obstruction with air retention and had central venous line insertion, all of which are risk factors for development of tension pneumothorax Tension pneumothroax requires immediate intercostal chest drain insertion.

**Figure 1:**
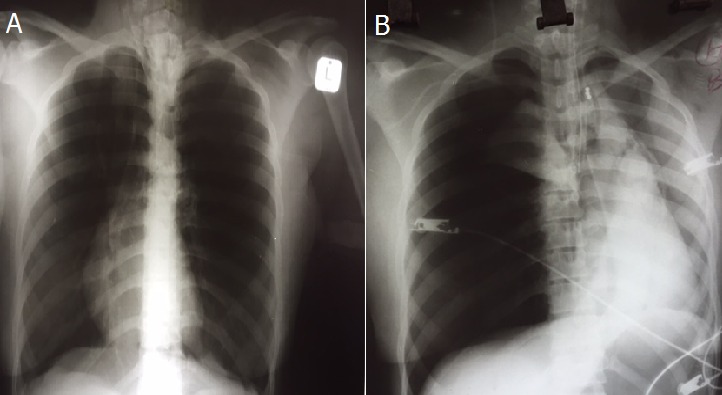
A): anterior Posterior bed side chest radiograph demonstrating dextrocardia with air trapping; B): tension pneumothorax displacing heart to left

